# Cancer Chemoprevention: Classic and Epigenetic Mechanisms Inhibiting Tumorigenesis. What Have We Learned So Far?

**DOI:** 10.3389/fonc.2018.00644

**Published:** 2018-12-21

**Authors:** Fabiana Henriques Machado de Melo, Julia Salles Oliveira, Viviani Olivastro Bressani Sartorelli, Wagner Ricardo Montor

**Affiliations:** Department of Physiological Sciences, Santa Casa de São Paulo School of Medical Sciences (FCMSCSP), São Paulo, Brazil

**Keywords:** chemoprevention, epigenetics, isoflavones, epigallocatechin gallate, resveratrol, sulforaphane, curcumin

## Abstract

Cancers derive from step by step processes which are differentiated by the progressively accumulated mutations. For some tumors there is a clear progressive advancement from benign lesions to malignancy and for these, preventive screening programs exist. In such cases having those benign lesions are a clear indicator of predisposition while for some other cases, familial patterns of cancer incidence and the identification of mutations are the main indicators of higher risk for having the disease. For patients identified as having predisposition, chemoprevention is a goal and in some cases a possibility. Chemoprevention is the use of any compound, either natural or synthetic that abrogates carcinogenesis or tumor progression, through different mechanisms, some of which have already been described. For example, the classic mechanisms may involve activation of free radical scavenging enzymes, control of chronic inflammation, and downregulation of specific signaling pathways. More recently, epigenetics allowed further understanding of the chemopreventive potential of several agents, such as sulforaphane, green tea derived compounds, resveratrol, isoflavones, and others which we exploit in this review article. Throughout the text we discuss the properties compounds should have in order to be classified as chemopreventive ones and the challenges in translational research in this area, as lots of the success achieved *in vitro* cannot be translated into the clinical settings, due to several different drawbacks, which include toxicity, cost, dose definition, patient adherence, and regimen of use.

## Carcinogenesis and Cancer Causing Agents

In order to understand the possibilities available to interrupt carcinogenesis and tumor progression, either through chemotherapy or chemoprevention, it is of vital importance to have a general view of the several steps necessary for tumor formation. Although different theories have been proposed, the most widely accepted one is that tumors arise from one single cell, that sequentially accumulates mutations and/or epigenetic alterations, loses control of proliferation and expands clonally, accumulating more and more modifications to allow the formation of the most advanced tumor form when one is referring to epithelial cell tumors or carcinomas, which is the potential metastatic cancer. As tumors expand accumulating mutations and epigenetic modifications, although it is a clonal expansion, the final mass is heterogeneous and keeps accumulating those which is the key to understand treatment resistance and abrupt aggressiveness change during treatment (1–4).

Carcinomas comprise the majority of solid tumors found in humans and they originate from cells that harbor mutations and epigenetic alterations which activate oncogenes, inactivate tumor suppressor genes, and at some level hinder DNA repair machinery, apoptosis mechanisms and in some cases even downregulates the immune system or creates mechanisms to evade it (1–3).

Throughout decades, single DNA modifications have been intentionally generated in laboratories with the intention of promoting carcinogenesis and tumor progression in cell culture models and what has been learned from these experiments was that no single modification is able to promote tumorigenesis, as there are complementary mechanisms that balance themselves when DNA changes occur, a collection of multiple modifications disturbing the systems mentioned above being necessary to make cells lose control of proliferation (5).

What causes those gene alterations are chemical, physical and biological agents, so we now know a plethora of cancer causing agents and their effects and can describe in details the effects of smoke compounds, x-rays and HPV to name a few on cell homeostasis disruption through DNA modification.

To the same extent we now understand that some tumors although harboring several different DNA modifications, have as the most striking contribution to its growth the ones that result in overexpression of some hormone receptor or the generation of truncation of any of the possible pathways downstream growth factor signaling, making it independent on proliferating signals from the microenvironment. Based on that, it is now common to treat and prevent breast and prostate cancers, for example, with hormone receptor inhibitors and specific molecules targeting mutated adaptors in cell signaling pathways have been developed in what we call target therapy (6).

Different from traditional chemotherapy, target therapy aims at acting specifically on cells harboring alterations and they fix the altered pathways on such cells or eliminate them (7).

Given the need for the tumor cells to accumulate gene alterations, as these have to disrupt different systems, such as those controlled by proto-oncogenes, tumor suppressor genes, DNA repair, apoptosis and others, it is expected that time is an important factor favoring tumorigenesis and that is why the elderly tend to have more cancer than younger people. Mutations and epigenetic alterations accumulate slowly, unless proliferation control has already been lost, when the proliferation speed does not allow cell cycle checkpoints to be verified and more and more modifications accumulate in each cycle (1, 5).

Thinking about the slow step by step events needed for tumor origin and progression, what allows carcinomas to progress from local confined tumors to widespread metastatic cancer is the ability to express enzymes for the purpose of degrading extracellular matrix proteins, so that they are not restricted to the epithelial layer but gain access to blood vessels and lymphatics from the underlying connective tissue. Only tumors expressing matrix metalloproteases, MMPs, have the ability to transition from the epithelium to the mesenchyme and spread out through the body (1–3, 5).

Although not valid for all of them, for some types of tumors, the slow transition from benign to malignant lesions can be observed and the typical DNA modifications found in one state or the other traced. That allows for some specific screening programs and early preventive interventions, such as happens for intestinal tumors that present themselves as benign lesions easily removed in colonoscopy procedures or cervical dysplasias found in pap smears and treated in a non-invasive way before they become fully installed cervical cancers (5).

Not only the existence of these pre-malignant lesions alerts patients for more attention and possible interventions but family patterns do so. Not all breast cancers in a family are an alert for special attention, but we know that those achieving younger women and presenting some mutations, such as in BRCA1 and 2 genes flag other close family women to pay more attention to preventive care. To the same extent, patterns of hereditary intestinal cancers can be present in different members of the same family. Even syndromes caused by deficient expression of tumor suppressor genes such as p53 and retinoblastoma (Rb) are described and carriers present several tumors throughout life (5, 8, 9).

That all being said gives us the clear notion that most tumors arise from random gene alterations and there is no way one can foresee them, but some cancers can be predicted. Patients have conditions that make them prone to tumorigenesis, either through genetic background and family patterns, environmental exposition of some type, lifestyle choices, and the existence of pre-malignant lesions or cancer promoting infections, making prevention a demand, if there is a way to do it.

## Chemoprevention

Chemoprevention is defined as the use of agents to inhibit tumorigenesis or tumor progression. The term is applied for agents that might have a role in any of the several steps mentioned above, either by blocking cancer causing agents and not allowing them to change DNA, increasing the role of the DNA repair system or acting on cells that carry DNA modifications already, decreasing cell cycle speed or even hindering events necessary for tumor spreading through metastasis. Therefore, chemoprevention can be used by people who have cancer already and also those that have a higher risk of developing it (10–13).

Chemopreventive agents cover a wide range of molecules from natural sources such as plants, fruits and vegetables to synthetic molecules, generally in use for other purposes and not cancer treatment. The identification of possible chemopreventive agents for study comes from different sources, but mainly: (a) observations that populations presenting some specific eating habits have lower incidence of specific cancers; (b) epidemiological studies or clinical tests of some drugs that show decreased number of cancers in some study populations as a secondary effect and (c) laboratory studies, when screenings show that the use of some molecules over tumor cell cultures not only inhibits cell proliferation but elicits surrogate markers of what we would call a malignant to normal reversion at some level (11).

Although the idea of chemoprevention is attractive and a large number of molecules have already been tested, in the United States there are only about 15 molecules approved by the FDA for use as chemopreventive agents (Table 1). Nonetheless, although the majority of tests to prove the chemopreventive property of potential molecules fail when brought from the laboratory to the population, a lot is learned in the study process and even when they are not approved, pathways involved in cell proliferation control are better understood (11).

**Table 1:** Examples of some approved agents for cancer risk reduction based on cancer type, mechanism of action and target population.

**Compound**	**Cancer type**	**Mechanism**	**Target population**
Tamoxifen	Breast	Selective estrogen receptor modulator	Women with ductal carcinoma *in situ* after surgery and radiation
Tamoxifen	Breast	Selective estrogen receptor modulator	Women at high risk for breast cancer
Raloxifene	Breast	Selective estrogen receptor modulator	Postmenopausal women at high risk for invasive breast cancer
Porfimer sodium + photodynamic therapy and omeprazole	Esophageal	Oxygen free radicals kills cancer cells after exposure to light	Barrett's esophagus patients
Aspirin	Colorectal	Inhibits COX and interrupts prostaglandin production	Adults ages 50–59 with higher risk for colorectal cancer
Celecoxib	Colorectal	Same as aspirin	Individuals 18+ from familial adenomatous polyposis families
Bacillus Calmette-Guerin (BCG)	Bladder	unknown	Carcinoma *in situ* of the urinary bladder
Valrubicin	Bladder	Interferes with DNA synthesis	Patients refractory to BCG
Fluoruracil	Skin	Interferes with DNA synthesis	Actinic keratosis
Diclofenac sodium 3%	Skin	unknown	Actinic keratosis
5-aminolevulinic acid + photodynamic therapy	Skin	Kills precancerous cells exposed to light	Actinic keratosis
Imiquimod	Skin	Enhances immune e response	Immunocompetent patients with actinic keratosis and warts
Ingenol mebutate	Skin	Induces primary necrosis	Actinic keratosis
HPV vaccine	Cervix, anus, and others	Prevents HPV infection through immune response	Varies from country to country but generally adolescents

In order to give examples of molecules that were put to test because of their chemopreventive potential, curcumin has been deeply studied as such, due to the low number of breast cancer cases in India. Several molecules derived from spices, teas, fruits and vegetables, from different sources such as lemon, saffron, garlic, broccoli, pomegranate, berries, and others have also been put to proof, due to the observational impression and epidemiological studies showing that people who have a diet richer in fruits and vegetables have lower incidence of cancer, the same thing happening to all possible vitamins and other micronutrients, especially the antioxidant ones, such as vitamin A, C, and E, but not only, as folate is in the list. Omega-3 fatty acids present in the Mediterranean diet are in that list too (14, 15).

For the same reason, drugs, studied for other purposes ended up in the list and the most studied ones are non-steroidal antiinflammatory drugs (NSAIDs), such as aspirin and selective COX-2 inhibitors, metformin used for diabetes and more recently statins used for cholesterol lowering (8, 16, 17). While for some drugs the evidences of chemoprevention are robust, such as for COX inhibitors and intestinal lesions, for others, such as metformin results are still controversial. *In vitro* and animal studies corroborate initial observational epidemiological studies, showing metformin induces G0/G1 cell cycle arrest, cell death through apoptotic and non-apoptotic pathways, decreased MMP-9 expression and activity, inhibiting cancer cell migration, which all indicate the possibility of chemoprevention and mechanistic models hypothesize the action being through decreased insulin levels, inhibiting IGF-1/IGFR-1 signaling or indirect action via energy depletion inhibiting PI3K/mTOR pathways, but when it comes to more controlled studies, as the level of stringency and control for confounding factors rises, from cohort and case-control studies to randomized clinical trials, the benefits almost disappear or are limited and not as striking (18, 19). Larger clinical trials, with better established surrogate endpoints, dose adjustments and longer observation time might bring the results of *in vitro* studies which are significant to the population, but those are still lacking.

Tamoxifen is the first chemopreventive agent approved by the FDA and it works because it is a selective antagonist of the estrogen receptor in breast. Given that a high proportion of breast cancers express estrogen receptors and depend on it for proliferation, the use of estrogen receptor antagonists has an important role in chemoprevention (20).

All of those present anti-tumoral effects when used over tumor cell cultures and revert the apparent tumoral phenotype at some level, but with the exception of NSAIDs preventing intestinal cancer recurrence or occurrence in high risk populations, the clear observation that the consumption of fruits and vegetables prevents several kinds of cancer, and that prevention of breast cancer recurrence benefits from the use of synthetic selective estrogen receptor modulators such as tamoxifen and raloxifen, no single molecule has been reported to be effective in humans and that might be because they exert their effects in specific tumors only and in higher concentrations or in combination with other molecules, or still their distribution through the body might hinder direct action on specific cells, which is not the case for tissue cultures in the lab (11).

Other than that it is very easy to observe a decrease in cell count or cell proliferation index in specific cells in the laboratory, but it is not the same when we extrapolate the assays to the population with the aim of preventing a disease that might not happen. A lot of discussion exists regarding the end points that should be observed *in vivo* and the corresponding surrogate markers (11).

It is important to consider also that although some individuals of the population might be identified as having a higher risk of developing cancer, it is not possible to know when that is going to happen and therefore, by definition, the use of chemoprevention lasts years and years and with that comes the need for the molecules to be the least toxic possible. No single molecule is exempt from toxicity depending on the dose and time of use and a serious risk/benefit consideration has to be made in order to decide on the approval of any molecule for such use (15).

Even safe medicines such as aspirin which is proved to be chemopreventive rise discussions on the proper doses and onset of use. People with higher risk of intestinal cancer benefit from the use of aspirin but discussions exist on the minimum dose and when it should start being used, since higher doses and continuous use might be related to gastric and intracranial hemorrhages. That makes it clear that it is not an adequate chemopreventive regimen for the general population but only for those at increased cancer risk. The possibility of local use or different administration routes has already been exploited in order to avoid systemic toxicity but there are no cases of success so far.

The ideal compound for chemoprevention is the one that has little or no toxicity, presents high efficacy in multiple sites, can be taken orally, the mechanism of action is known, is a low cost drug and has easy human acceptance as it will have to be taken for years.

Omega-3 fatty acids are another example of molecules that seem to be harmless and work *in vitro*, inhibiting tumor growth but lack evidence in populational studies and have the potential to cause harm if proper use is not made, since it is free fatty acids in the circulation that can render cells less sensitive to insulin and thus promote hyperglycemia (21).

Dosing is of uttermost importance. Studies show that multivitamins prevent some types of cancer when used by populations that present deficiencies, but the opposite might happen in supraphysiological doses. In general, no benefits are found and no chemopreventive properties have been reported due to the use of multivitamins or any vitamin alone when no deficiency is present, but several studies report an excess of tumors, especially lung tumors in high risk populations supplemented with beta-carotene and other vitamin A precursors. The Alpha-Tocopherol and Beta-Carotene (ATBC) trial in northern Europe reported an increased incidence of 18% for lung cancer among those receiving β-carotene. The Beta-Carotene and Retinol Efficacy Trial (CARET) in the United States showed an increased incidence of 28% for lung cancer on the β-carotene and retinol arm of the study (22–24).

The difficulty in translational medicine, meaning bringing *in vitro* discoveries to the bed side is very significant when plants and plant derived products are studied. Most plants have biologically active compounds, such as alkaloids, terpenes, lecithins, and flavonoids, among others and several of those possibly have antitumor and cytotoxic effects. But finding such molecules either as an isolated product or in plant extracts which are not toxic for continuous human consumption is a challenge.

What has not been difficult to prove so far are the benefits from diet and lifestyle modification targeting cancer prevention. Physical activity, weight control, low fat diet, consumption of abundant fruits and vegetables and smoke cessation have all been proved as tools to prevent cancer. No isolated compounds from fruits and vegetables have proved any benefit similar to what the consumption of fruits and vegetables promotes. It might be that the molecules tested are not the ones responsible for prevention or it only acts when in combination with others, which might be the case as we have different tumorigenesis mechanisms that might need to be hindered by multiple chemopreventive agents at the same time (25–27).

## Classic Molecular Mechanisms Involved in Chemoprevention

Surprisingly for most of the putative or approved chemopreventive agents the mechanism involved is not fully understood. A special mechanistic class comprises compounds that circumvent DNA damage by direct inactivation of carcinogens either acting as free radical scavengers or inducing enzymes involved in scavenging. Other than that, a plethora of molecules can exert this antitumor role by acting as anti-inflammatory compounds. Inflammation can trigger ROS formation and therefore DNA alterations, but it also plays an important role in tumor progression as NFκB, for example, an important inflammatory molecule that acts on the promoter of several genes involved in cell proliferation. That is one of the reasons why obesity is considered a cancer promoting condition, due to the inflammatory state of adipose tissue in obese people (28).

For example, curcumin and green tea derived molecules have been showed to stabilize IκB, the cytoplasmic inhibitor of NFκB, avoiding its translocation to the nucleus, where it acts as a pro-inflammatory molecule, since these compounds inhibit the proteasome, responsible for degrading IκB (29, 30).

*In vitro* studies show that established breast cancer cell lines when treated with polyunsaturated fatty acids present proliferation inhibition, followed by apoptosis, usually accompanied by the formation of reactive oxygen species. The mechanism by which proliferation inhibition occurs is not clear, but one of the hypothesis is the replacement of lipids from the cell membranes by the supplemented lipids or their derivatives, which when metabolized by cyclooxygenase (COX) generate different products, named resolvins which are different from the products generated when arachidonic acid is metabolized. Thus, proinflammatory cascades initiated by the production of prostaglandins would be interrupted by the lack of substrate, changing the destiny of the cell in relation to its participation in inflammatory and or tumorigenic processes (21).

Transcription factors responsible for the expression of enzymes that promote free radical scavenging are induced not only during oxidative stress but also due to the presence of several different phytochemicals, of which maybe sulforaphane derived from cruciferous vegetables such as broccoli and resveratrol derived from grapes and grape products are the most important ones. One of such transcriptions factors is called NRF2 (NFE2-related factor 2), the most important regulator of antioxidant defenses, binding to antioxidant responsive elements in the promoter of several genes that code for enzymes such as glutathione S-transferase, NADPH:quinone oxidoreductase and others (31, 32).

Several phytochemicals induce cell death through autophagy, such as resveratrol, apigenin found in apples and several other fruits and vegetables, curcumin, fisetin, quercetin, [6]-gingerol, piperlonguminine, and others. Apoptosis is also a pathway commonly activated by such phytochemicals and each seems to activate either autophagy or apoptosis through different mechanisms (33).

Specific studies aiming at identifying compounds that would inhibit tumor cells from epithelial-mesenchymal transition, an important step for metastasis generation, found important phytochemicals such as silibinin, curcumin, green tea derived compounds, [6]-gingerol, resveratrol and others to induce increased expression of E-cadherin, a molecule responsible for maintaining cells attached to each other, making it difficult for them to migrate in adjacent tissue invasion (34–36).

**Figure 1:**
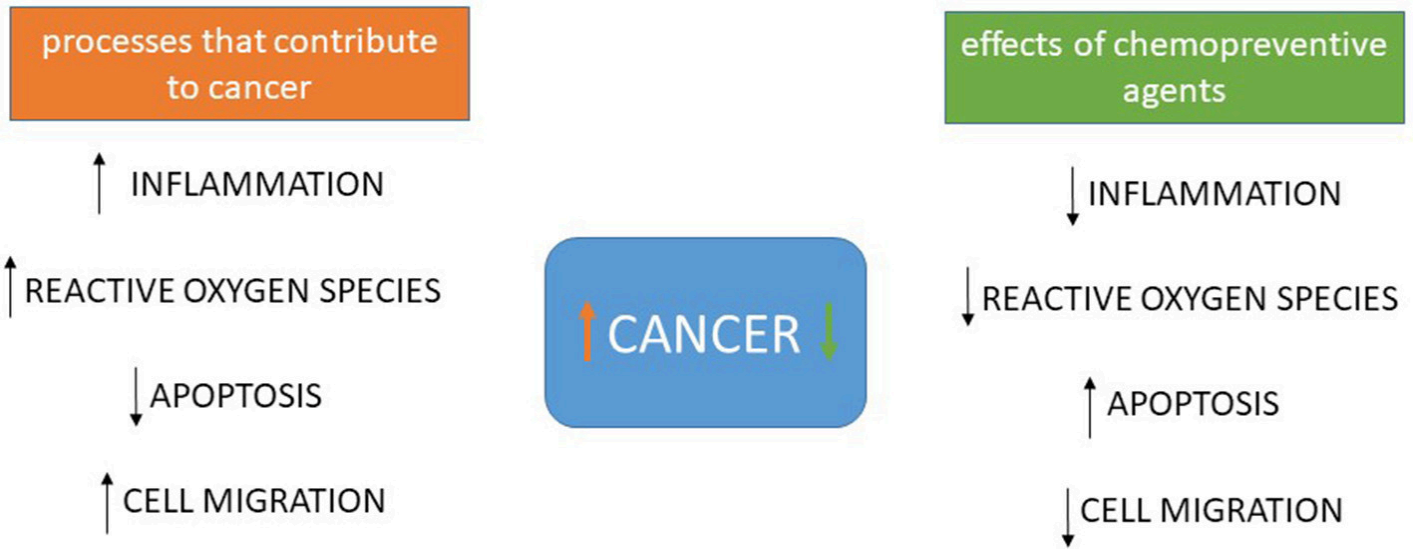
Processes that contribute to tumorigenesis and cancer progression and the effects of chemopreventive agents. Most chemopreventive agents act in more than one process, but overall it is well-established that curcumin, polyunsaturated fatty acids, and green tea compounds decrease inflammation, several including resveratrol and sulforaphane decrease reactive oxygen species, resveratrol, apigenin, curcumin, fisetin, quercetin, [6]-gingerol and piperlonguminine promote apoptosis and autophagy, some of those also decreasing inflammation and silibinin, curcumin, green tea compounds, [6]-gingerol and resveratrol have already been shown to increase E-caderin expression and decrease MMP-9 expression and activity, inhibiting cell migration and epithelial-mesenchymal transition. Several other compounds promote similar effects but these are the best described ones, whose effects are direct or indirect but affecting those processes.

Also acting by inhibiting epithelial-mesenchymal transition, now through inhibition of expression of MMPs, mainly MMP-9, phytochemicals such as curcumin, [6]-gingerol, luteolin and others have been described *in vitro* (37–40). A summary of the possible targets of chemopreventive agents is seen in Figure 1.

The role of different viruses and even bacteria promoting carcinogenesis, such as hepatitis B and C virus, HIV, HPV, and *Helicobacter pylori* are clear and the mechanism identified. The development of vaccines against those and early treatment to prevent their carcinogenic mechanism can be considered a chemopreventive action (41).

While such mechanisms become more and more clear through *in vitro* studies, the great recent development of Epigenetics shed some light into possible mechanisms involved in chemoprevention, shifting from the classic free radical scavenging, inflammation control, and others described above. Below we described the state of the art of Epigenetics and the current understanding of chemoprevention, detailing some of the mechanisms already described for some specific agents.

## Chemoprevention Explained Through Epigenetics

Epigenetic modifications also contribute to abnormal gene expression and consequently to malignant transformation and tumor progression. Epigenetics controls gene expression through chromatin remodeling without any change in gene sequence (42), regulating fundamental biological processes such as embryological development, differentiation, inactivation of the X chromosome, genomic imprinting and specific tissue expression (43). Chromatin can be organized as heterochromatin, which is highly condensed and transcriptionally inactive or as euchromatin, which is less compacted and contains the most transcribed genes (44, 45). Several epigenetic mechanisms are involved in the nucleosomes organization and have been described to be altered during cancer development, including DNA methylation, histone post-translational modifications, such as methylation, acetylation, phosphorylation and nucleosomes occupancy dynamics (42, 43, 46).

**Figure 2:**
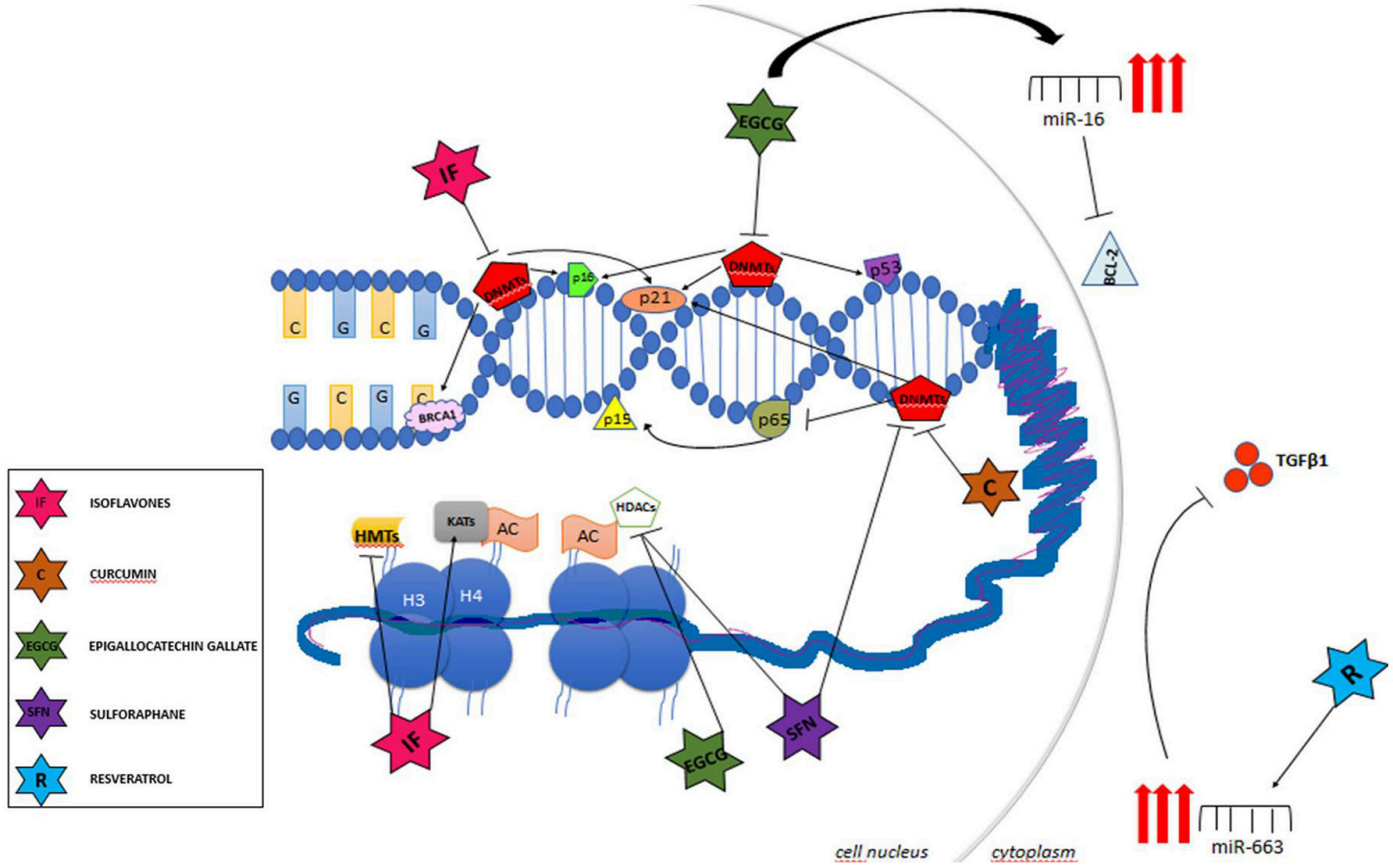
Main targets of chemopreventive agents acting as epigenetic regulators.

Unlike genetic alterations, epigenetic mechanisms are potentially reversible and can be modulated by the fluctuation of environmental factors such as nutrition, oxidative stress, pollution, inflammation, and life style (43, 47). Therefore, a growing body of evidence has shown the potential of the epigenetic therapy in many types of cancers (42, 48, 49). Currently, the DNMT inhibitors 5-azacytidine (5-AZA) and 2′-deoxy-5-azacytidine (DAC), have been approved by the FDA for the treatment of myelodysplasia myelodysplastic syndrome (MDS), a preleukemic syndrome and myelomonocytic leukemia, and the results for solid tumor therapy are promising (42, 48, 49). Additionally, it has been described that nutrients and bioactive foods can change gene expression through regulation of different epigenetic mechanisms in many ways. DNA and histone methylation can be modified by folate, vitamin B12, methionine, betaine, and choline by altering 1-carbon metabolism while resveratrol, genistein, and curcumin inhibit enzymes involved in chromatin modifications as will be described below (Table 2). In this way, certain bioactive food components can be used as chemopreventive agents modulating many aspects of tumor development (Figure 2).

**Table 2:** Mechanism of action of the different bioactive compounds for which epigenetic alterations have been described.

**Mechanism of action**	**Neoplasia**	**Study type**	**Target genes**
**ISOFLAVONES**			
Decreased methylation	Breast cancer	*In vitro*	BRCA1, BRCA2, ATM, APC, PTEN; SERPINB5
Increased acetylation	Breast cancer	Preclinical studies: *in vitro* and *in vivo*	ERα
Increased active mark H3K4 and acetylation	Breast cancer	*In vitro* and *in vivo* (xenografts)	p16^INK4a^; p21^WAF1^
Increased repressive mark H3K27	Uterine leiomyomas	Human	p16^INK4a^; p21^WAF1^
Increased acetylation, decreased methylation and increased active mark H3K4	Prostate cancer	*In vitro*	p16^INK4a^; p21^WAF1^, BTG3, AKT, CYLD
**EPIGALLOCATECHIN GALLATE**			
Decreased methylation	Breast, colorectal and skin cancer	*In vitro*	p16^INK4a^; p21^WAF1^, RARβ, RXRα, MGMT, MLH1
Decreased methylation	Oral squamous carcinoma	*In vitro*	RECK
Increased tumor suppressor miR	Hepatocarcinoma	*In vitro*	BCL-2
Decreased oncomir	NSCLC	*In vivo*	p53
**RESVERATROL**			
Decreased oncomiRs	Colorectal cancer	*In vitro*	TGFβ1
Incresead methylation	Breast cancer	*In vitro*	AURKA PLK1
Increased tumor suppressor miRs	Colorectal cancer	*In vitro*	E2F3
Hyperacetylation	Breast cancer	*In vivo*	BRCA1
**SULFORAPHANE**			
Hyperacetylation	Colorectal and prostate cancer	*In vitro* *In vivo*	BAX p21
Hyperacetylation	Breast cancer	*In vitro*	Caspases Cytochrome c
Decreased methylation	Breast and prostate cancer	*In vitro*	PTEN RARβ2
Hyperacetylation Decreased repressive mark	Breast cancer	*In vivo*	hTERT
**CURCUMIN**			
Decreased methylation	Colorectal cancer	*In vitro*	NF-κB pathway
Decreased methylation	Cervical cancer	*In vitro*	RARβ2
Decreased methylation	Myeloid leukemia	*In vitro* *In vivo*	p15I^NK4B^
Increased suppressor miRs Decreased oncomiRs	Colorectal cancer	*In vitro*	Cyclin D1 and E1 CDK4 and 6 cMYC
Increased suppressor miRs Decreased oncomiRs	Melanoma	*In vivo*	BCL-2 PCNA
Increased suppressor miRs	Pancreatic carcinoma	*In vivo*	Notch1 MMp-9

## DNA Methylation

In mammalian cells, DNA methylation takes place only at cytosine bases at CpG dinucleotides and is catalyzed by DNA methyltransferases (DNMTs), which transfer the methyl group from S-adenosyl-L-methionine to the cytosine residues. DNMT1 binds preferentially to hemimethylated DNA during DNA replication and then methylates CpG dinucleotides in newly synthesized DNA strands maintaining the methylation pattern of the parental strand, while the main function of the *de novo* DNMTs 3a, 3b, and L are to establish a new methylation profile. DNA demethylation in mammalians is catalyzed by the ten-eleven translocation (TET1-3) family of proteins, DNA hydroxylases that promote the conversion of 5mC to 5hmC and further oxidation of 5hmC, resulting in the derivatives 5-formylcytosine (5fC) and 5-carboxylcytosine (5caC) and transcriptional regulation through reversal of DNA methylation patterns (50, 51). Mutations in DNMTs related to human diseases, including cancer, are extremely rare (52, 53). However, sequencing of cancer genomes has identified mutations in DNMT3A in ~25% of patients with acute myeloid leukemia (AML), decreasing the catalytic activity of the enzyme (51). These somatic mutations are also found in myeloproliferative diseases (MPD) and myelodysplastic syndromes (MDS). A high prevalence of TET2 mutations was described in patients with chronic myelomonocytic leukemia (CMML), AML, MPD, and MDS (54). Aberrant DNA methylation patterns are frequently found in cancer cells, where DNA hypermethylation is present in CpG islands of tumor suppressor gene promoters, invariably inhibiting their transcription and global DNA hypomethylation is found in repetitive elements contributing to genomic instability, loss of imprinting and oncogene activation (46). Interestingly, DNA hypermethylation also inhibits the expression of innumerous non-coding RNAs with anti-tumoral activity (42). On the other hand, it was shown that many actively transcribed genes have high levels of CpG methylation within the gene body, indicating that the methylation site is fundamental to its role in transcriptional regulation (42). It was described that almost all tumors harbor innumerous genes with abnormal methylation profile, driving tumorigenesis through regulation of proliferation, survival, and apoptosis.

## Histone Post-translational Modifications

Histones can be modified by several post-translational modifications, including methylation, acetylation, ubiquitination, phosphorylation, sumoylation, biotinylation, and ADP-ribosylation (42). The site of histone alterations is at the histone tail that contains from 15 to 38 aminoacids, the lysine residues being either methylated (mono-, di-, and tri-) or acetylated, and arginine residues mono- or di-methylated. In general, histone lysine acetylation is associated with gene activation while histone lysine methylation can be a mark for both transcriptionally permissive patterns, in the case of H3K4 trimethylation and transcriptionally repressive patterns, as in the case of tri-methylated lysine 27 on histone 3 (H3K27) and H3K9 trimethylation chromatin modifications (42). Histone acetylation patterns are maintained by histone lysine acetyltransferases (KATs) and histone deacetylases (HDACs) and histone methylation status by histone methyltransferases and histone demethylases (55, 56). It is important to note that these histones alterations are dynamic and are changing depending on cellular demand. Furthermore, active and repressive marks are not always mutually exclusive, representing “bivalent domains,” meaning that histones mark combination will determine the chromatin organization and consequently gene pattern expression. Several enzymes responsible for histones modifications are altered and implicated in carcinogenesis. Several examples have been described, including recurrent chromosomal translocations, coding mutations and overexpression in different types of neoplasias as AML, ALL, and breast, colorectal and pancreatic carcinomas (57). Another issue that must be taken into account is the fact that DNA methylation and post-translational histone modifications are associated, since HDACs are recruited to sites of hypermethylated DNA together with methyl-cytosine binding proteins to the maintenance of transcription repression (45, 57).

## Chromatin Remodeling Complexes

Together with covalent modifications of DNA and histone tails, enzymes are recruited to chromatin and cooperate to promote nucleosomes remodeling in an ATP-dependent manner regulating gene expression. ATP-dependent chromatin remodelers have a common ATPase domain and the free energy derived from ATP hydrolysis allows the machinery to reorganize the chromatin. These protein complexes are divided into families based on the subunit composition and biochemical activity, including SWI/SNF, ISWI, INO80, SWR1, and NURD/Mi2/CHD complexes (58, 59). The alterations in the expression of these complexes are associated with tumor development and SWI/SNF are emerging as tumor suppressors, since inactivating mutations have been described in many types of cancers (50).

Other classes of chromatin remodeling complexes are the Trithorax-group (trxG) and Polycomb-group proteins (PcG) that have opposite actions in the dynamics of chromatin organization. TrxG proteins maintain the active state of gene expression while the PcG proteins counteract this activation with a transcriptional repression (60). Polycomb group proteins are composed by large multimeric complexes of two types, polycomb repressive complex 1 (PRC1) and PRC2. PRC2 is comprised by four subunits, SUZ12 (zinc finger), EED, EZH1, or EZH2 (SET domain with histone methyltransferase activity) and RbAp48 (histone binding domain) and has the primary function of trimethylating histone H3 on lysine 27 (H3K27me3), a mark of transcriptionally inactive chromatin. PRC1 contains the Bmi-1 protein, that binds to the trimethylated H3K27 and catalyzes the ubiquitination of histone H2A on lysine 119 (H2AK119Ub1), stabilizing the repressive state. The Trithorax complex activates gene transcription by promoting the trimethylation of lysine 4 of histone H3 (H3K4me3) at specific sites in chromatin recognized by the complex (61). Alterations in the expression and activity of polycomb proteins as EZH2 and MLL have been associated with the development of many cancers, including AML, ALL, and multiple myeloma (62).

## Micro-RNA

Micro-RNAs are endogenous, small non-coding RNAs, about 22 nucleotides in length that regulate gene expression by targeting a specific mRNA, binding to it and inhibiting its expression by a variety of mechanisms. Translational repression as a consequence of miRNA binding to the 3′UTR of the mRNA is the main mechanism; however, mRNA degradation and destabilization have also been showed. MiRNAs have innumerous targets and modulate the expression of multiple genes, in this way, these regulate important biological processes such as proliferation, survival, differentiation and apoptosis (63, 64). Therefore, miRNA deregulation is involved in the pathogenesis of several diseases, including cancer. In tumorigenesis, microRNAs can act as either oncogenes or tumor suppressors, contributing to cancer development and modulating the hallmarks of cancer, such as sustaining proliferative signaling, resisting cell death, inducing angiogenesis, activating metastasis, reprogramming energy metabolism, and promoting evasion of the immune system (65).

Importantly, all these epigenetic mechanisms work in association with methyl-cytosine-binding proteins and chromatin remodeling complexes to promote and to maintain the dynamic states of the chromatin between nucleosomes, in a compacted and transcriptionally silent state or in an open and transcriptionally active state according to cell's requirements.

## Examples of Compounds for Which Epigenetic Chemopreventive Mechanism Are Described

### Isoflavones

Some types of isoflavones (IF) are closely related to phytoestrogens and are predominantly found as glycoside conjugates. The most prevalent dietary IF include genistein (GEN), daidzein (DAI), and glycitein (GLY), being the most common sources soybeans and soy-based foodstuffs. In addition to estrogen receptor signaling modulation, antioxidant activity and innumerous anti-tumoral activities have been shown in breast tumor cells by inhibiting receptor tyrosine kinase signaling pathways (66, 67), IF have the capability of regulating the epigenome, leading to chromatin remodeling, through differential recruitment of enzymes of the epigenetic machinery to the promoter of regulated genes (68, 69). *In vitro* studies showed that IF inhibited the expression and activity of DNMTs 1, 3a, and 3b and consequently decreased methylation of the promoters of tumor suppressor genes, leading to alteration in the expression of different proteins, including BRCA1 and BRCA2, involved in DNA repair mechanisms; ATM (ataxia telangiectasia mutated), an important cell cycle checkpoint kinase; APC (adenomatous polyposis coli), an antagonist of the Wnt-signaling pathway; the phosphatase PTEN, inhibitor of the PI3K-AKT signaling pathway and SERPINB5 (mammary serpin peptidase inhibitor) in breast tumor cells (70, 71). Preclinical studies also showed the involvement of GEN in regulation of histone modifications. In *in vitro* and *in vivo* preclinical studies, GEN reactivated ERα expression through epigenetic mechanisms in ERα-negative breast cancer cells, increasing cancer cells sensitivity to apoptosis and consequently TAM-mediated estrogen-dependent therapy. In ERα-negative breast cancer cells, GEN inhibits the binding of DNMT1 and HDAC to ERα promoter (72). Treatment of precancerous and breast cancer cells with GEN increased the expression of tumor suppressor genes *p16*^*INK*4*a*^ (*p16*) and *p21*^*WAF*1^ (*p21*) through enrichment of transcriptional active markers such as acetyl-H3, acetyl-H4, and trimethyl-H3K4. Moreover, dietary administration of GEN suppressed the growth of breast cancer xenografts (73). Interestingly, in this work the authors suggested that GEN would be more effective in chemoprevention than in chemotherapy, since lower doses of GEN were sufficient to induce the death of precancerous breast cells. This result is in agreement with extensive data from the literature that shows that women from Asia, where there is a higher intake of soy-based foods, have lower incidence of breast cancer, suggesting the participation of IF in epigenetic reprogramming of breast epithelial cells. Several clinical trials are under investigation, evaluating the efficiency of GEN as a single agent or in combination with other drugs in the treatment of breast cancer. On the other hand, a recent epidemiological study showed a positive correlation between early life soy consumption and increased risk of uterine leiomyomas (74). The increased incidence of leiomyomas seems to be associated to augmented expression of estrogen-responsive genes induced by decreased EZH2 activity and levels of H3K27me3 on promoters of these genes (75). Genistein also seems to be effective in the prevention of prostate cancer since it decreased androgen receptor (AR) expression in prostate cancer cells through inhibition of HDAC6-Hsp90 interaction. AR protein is degraded via an ubiquinin-proteasome pathway which is prevented by stabilization with the heat shock protein Hsp90, which in turn is inactivated by acetylation. Genistein decreases HDAC6 expression, abrogating Hsp90 activity, and consequently downregulating AR and prostate cancer cell proliferation (76). Moreover, GEN treatment increases the expression of several KATs, augmenting the acetylation of histones H3 and H4 at the promoters of specific tumor suppressor genes and consequently their reexpression, including p16, p21, and BTG3 (B-Cell translocation gene 3), a negative regulator of E2F-1 signaling, in prostate cancer cell lines (69, 77). The increased transcription of BTG3 induced by GEN was also a result of activating H3K4me2 and H3K4me3 marks and decreased methylation at the BTG3 promoter (69). The expression of other tumor suppressor genes, such as PTEN, a PI3K/AKT signaling pathway inhibitor, and CYLD, an NF-κB inhibitor, is restored after GEN treatment because of increased H3K9 acetylation and decreased H3K9 methylation at their promoters (78). Therefore, GEN treatment reduced AKT activation and proliferation and migration of prostate cancer cells. Genistein treatment is also associated with oncomiRs and tumor suppressor miRs reexpression in prostate cancer cells (79–81). In other tissues, including kidney, colon, and pancreas, several data also showed the association between genistein and epigenetic modifications (82–86).

### Epigallocatechin Gallate

Epigallocatechin gallate (EGCG), also known as epigallocatechin-3-gallate, is the ester of epigallocatechin and gallic acid, being the type of catechin most abundant in green tea. Besides its role as an antioxidant and anti-inflammatory molecule, EGCG has the capability of regulating gene expression through modification of epigenetic machinery activity; therefore, EGCG is a potential anti-tumorigenic compound. Several data have showed that EGCG treatment inhibited DNMTs expression and activity, inducing promoter hypomethylation of important tumor suppressor genes, including p16, p21, RARβ, RXRα, MGMT, and MLH1, and inhibiting proliferation and inducing apoptosis of different cancer cell lineages (87–91). EGCG treatment decreased RXRα promoter methylation and consequently restored the expression of RXRα protein in human colon cancer cell lines, decreasing cell proliferation. RXRα is a nuclear transcription factor that is normally downregulated in several tumors, including colon carcinoma, therefore, EGCG therapy can be seen as a chemoprevention strategy in cancer (91). EGCG also seems to be effective in more advanced stages of tumor progression. EGCG treatment of human oral squamous cell carcinoma cell lines partially reversed the hypermethylation status of the *RECK* gene, a matrix metalloproteinase inhibitor, and increased RECK expression, consequently, extracellular matrix degradation was inhibited and cell invasion was suppressed (92). Epigenetic alterations are normally found in precancerous lesions, so the reexpression of tumor suppressor genes such as p16 and p21 that control cell cycle progression or MGMT and MLH1 that are part of the DNA repair machinery contributes to cell homeostasis, so these bioactive components can be considered important chemopreventives agents. In human skin carcinoma cells, the EGCG treatment inhibited the activity of HDAC in addition to the DNMT activity, since the levels of acetylation of H3 and H4 were decreased. Moreover, EGCG reduced the levels of methylated H3-Lys 9, a mark of repressed transcription (90). EGCG is also associated with alteration in the expression of miRNAs. In human hepatocellular carcinoma HepG2 cells, treatment with EGCG induced upregulation of 13 and downregulation of 48 miRNAs (93). The tumor suppressor miR-16 is one of the miRNA increased, which in turn, induces apoptosis by targeting and repressing the expression of the anti-apoptotic protein BCL-2 (93). In addition, the combination of EGCG with different chemotherapeutics improves the efficiency of the treatment in preclinical studies in several cancer models (94–97). The combination of EGCG and anti-HER2 therapies, namely pertuzumab or temsirolimus, abrogates anti-HER2 drug resistance in breast cancer models *in vitro* and *in vivo*, showing the role of EGCG not only in chemoprevention, but also as a therapeutic agent and suggesting the involvement of the epigenome reprogramming in acquired resistance (95). EGCG therapy increased the efficiency of cisplatin treatment *in vivo*, in a non-small cell lung cancer model. The tumor growth inhibition promoted by EGCG is associated with downregulation of hsa-miR-98-5p, followed by an increase in p53 expression (97). Moreover, EGCG overcomes acquired resistance to 5-fluorouracil (5FU), the standard chemotherapeutic drug in colorectal cancer by inducing apoptosis and cell cycle arrest in 5FU-resistant colorectal cancer cells and by inhibiting the self-renewal of cancer stem cells *in vitro* and *in vivo* (94). EGCG suppressed cancer stem cell generation by decreasing the expression of the subunits of the polycomb repressive complex, EZH2, and SUZ12, and the levels of tumor suppressor miRNAs miR-34a, miR-145, and miR-200c.

### Resveratrol

Resveratrol is a stilbenoid, a type of natural phenol, and a phytoalexin produced by several plants, including grapes, peanuts, cranberries, blueberries, and mulberries. Resveratrol consumption has been associated with low incidence of diabetes, cardiovascular and inflammatory diseases (98, 99). Several publications have showed the anti-inflammatory effects of resveratrol through decreased expression of transcription factors associated with inflammation such as NFκB or activator protein-1 (AP-1) components c-Jun and c-Fos and the inhibition of the secretion of pro-inflammatory mediators such as prostaglandin E2 (100, 101). These effects are also associated with the anti-tumoral activity of resveratrol since chronic inflammation is associated with malignant transformation and tumor progression. It has been recently demonstrated that resveratrol can modulate inflammation through epigenetic mechanisms. Resveratrol induced the upregulation of *miR-663*, a microRNA associated with immune response modulation, in human monocytic cells, which in turn, decreased AP-1 activity and impaired the increase of the oncomiR pro-inflammatory *miR-155* (102). Resveratrol also increased the expression of tumor suppressor microRNAs in cancer cells, resulting in growth inhibition (96, 103). In human colon cancer cells, resveratrol caused the upregulation of *miR-34a*, causing the reduction of the target gene E2F3 and its downstream adaptor Sirt1. Furthermore, resveratrol increased the efficacy of 5-FU, resulting in a synergistic growth impairment and decreased apoptosis resistance (96). In addition, resveratrol treatment decreased the levels of miRNAs frequently overexpressed in colorectal cancer, including *miR-17, miR-21, miR-25, miR-26a, miR-92a-2, miR-103-1*, and *-103-2*, or *miR-181a2* and upregulated the tumor suppressor *miR-663, reducing* TGFβ1 expression and the downstream activation of SMADs (103). Resveratrol impaired the proliferation of breast cancer cells by inducing the hypermethylation of the promoters of the genes of Aurora protein kinase (AURKA) and the Polo-like kinase-1 (PLK1) and consequently decreased its expression (104), reinforcing the potential role of resveratrol in cancer therapy. Resveratrol also abolished the histone modifications induced by the activation of the aromatic hydrocarbon receptor which is associated with the development with several tumors. Treatment of breast cancer cells with resveratrol increased acetylation of H3 and H4 decreased methylation of H3K9 and recruited MBD2 to the BRCA-1 promoter (93, 105). In human hepatoblastoma cell lines resveratrol also impaired HDAC activity and increased acetylation of H3, resulting in cell proliferation inhibition (106).

### Sulforaphane

Sulforaphane (SFN) is a type of isothiocyanate and belongs to the group of organosulfur compounds. It is derived from cruciferous vegetables such as cabbages, broccoli, broccoli sprouts, and brussels sprouts. In broccoli and broccoli sprouts, SFN is found as the glucosinolate precursor glucoraphanin that is converted to SFN by microbial hydrolases present in gut bacteria or plant myrosinases. Several studies have shown that SFN exerts anti-tumor activities through multiple mechanisms, resulting in apoptosis induction, growth inhibition and migration and invasion abrogation (107–110). In addition, SFN has the capability of inhibiting cancer development by modulating epigenetic mechanisms as demonstrated by *in vitro* and *in vivo* studies. SFN inhibited HDAC activity, resulting in increased global histone acetylation and apoptosis induction in different tumor cell lines (111–113). In human prostate and colon cancer cells, the increased expression of p21 and Bax was associated with hyperacetylation of histone H4 at their promoters (111, 112). SFN administration also decreased the HDAC activity *in vivo*, with concomitant increase in acetylated histones H3 and H4, suppressing tumor development and implying the therapeutic role of SFN in cancer (114, 115). In human breast cancer cells, induction of apoptosis by SFN is associated with increased expression of caspase, cytochrome c release into the cytosol and poly (ADP-ribose) polymerase cleavage (113). Moreover, SFN treatment reactivated the expression of tumor suppressor genes aberrantly methylated through DNMTs inhibition in human prostate and breast cancer cell lines (116, 117). Additionally, it has been showed that besides inducing the acetylation of histones H3 and H4 in hTERT promoter, SFN treatment also decreased the levels of inactive chromatin markers trimethyl-H3K9 and trimethyl-H3K27 in human breast cancer cells in a dose-dependent manner (118).

### Curcumin

Curcumin, a component of the popular Indian spice, is a diarylheptanoid, belonging to the group of curcuminoids, which are natural phenols, being the main curcuminoid of turmeric (*Curcuma longa*), a member of the ginger family, *Zingiberaceae*, a rhizomatous herbaceous perennial plant. Traditionally, curcumin has been used as an anti-inflammatory compound, since it inhibits NF-kB activity and reduced cyclooxygenase 2 and prostaglandins production (119). The anti-tumoral activities of curcumin are mediated through inhibition of multiple signaling pathways involved in regulation of proliferation, apoptosis, survival, angiogenesis and metastasis (119–124). Furthermore, curcumin seems to modulate gene expression through epigenetic mechanisms, implicating curcumin as a potential chemoprevention agent. Curcumin inhibited DNMTs activity and altered the methylation pattern in different tumor cell lines (125–128). In human colon cancer cells, curcumin treatment is not associated with global DNA hypomethylation as changes in methylation status of long interspersed nuclear elements-1 (LINE-1) were not found. Otherwise, curcumin decreased the methylation pattern of genes related to the NF-κB pathway (125). In human cervical cancer cell lines, curcumin treatment resulted in demethylation of the tumor suppressor RARβ2 gene promoter and induced its repression (126). In AML cell lines curcumin downregulates DNMT1 expression by decreasing the binding of the transcription factors p65 and Sp1, with concomitant reexpression of the tumor suppressor gene *p15*^*INK*4*B*^ due the demethylation of its promoter. Moreover, curcumin induced the G1 cell cycle arrest tumor cell apoptosis *in vitro*. *In vivo*, curcumin decreased tumor cell growth, showing the potential value of curcumin as a therapeutic drug (129). It was also demonstrated that curcumin regulates key cancer-associated micro-RNAs (130–132). Curcumin induced downregulation of the oncogenic miR-27a and upregulation of tumor-suppressive miR-34a in colorectal cancer cells and affect the expression of their downstream targets, leading to cell cycle arrest and apoptosis (130). In a melanoma model *in vivo*, it has been showed that curcumin administration altered miRNA signature of engrafting melanoma, showing an upregulation of tumor suppressive micro-RNAs and decreased levels of oncomirs, being the mmu-miR-205-5p the most significantly altered (131). Between the putative targets of the altered micro-RNAs, anti-apoptotic (BCL-2) and proliferating cell nuclear antigen (PCNA) were significantly downregulated in curcumin-treated tumors. In another study, curcumin was associated with reduced expression of EZH2 and upregulation of a panel of tumor-suppressive microRNAs, such as *let-7a,b,c,d, miR-26a, miR-101, miR-146a*, and *miR-200b,c* that are normally absent in pancreatic carcinomas. Moreover, curcumin administration inhibited pancreatic cancer tumor growth (132).

## Conclusion and Future Directions

As we understand carcinogenesis and tumor progression more and more we identify individuals who are at increased risk of developing cancer. Identifying those individuals, the obvious interest is to be able to prevent the disease somehow and to do so, a plethora of compounds have been tested and used over the last decades. One of the premises of chemoprevention is understanding the mechanism of action through which the compounds exert their role and that is not the case for most of the compounds we know. Some are partially understood and for some the mechanism is completely unknown. Epigenetics has been complementing what is known about chemoprevention mechanisms and opening up possibilities for the discovery of new candidate molecules.

The goal of the new era of cancer treatment has been the mapping of key components of signaling pathways involved in tumorigenesis and the development of innumerous drugs to be used in target therapy (7). Additionally, the combination of different kinds of therapy, including immunotherapy has improved the clinical outcomes. It is important to extend this knowledge to the chemoprevention area, characterizing molecular alterations of patients with a great risk of developing cancers, e.g., familial cancers, and targeting simultaneous, for example, histone modifications and DNA methylation, leading synergistically to the reactivation of aberrantly silenced genes.

As for the population, what can be recommended so far is a diet rich in fruits and vegetables as different phytochemicals definitely play a complementary role in cancer prevention. Although, there is no definition of dose and complete mechanistic understanding it is more and more obvious that what we eat is important to modulate our cancer risk. Most probably it is not one single compound or one single food that will have the power to change this risk, but the daily combination of what is known to have anti-cancer potential plus the avoidance of what is known to increase cancer risk that, in what we clearly define as a healthy balanced diet will directly protect individuals.

For the researchers, the next step would be improving *in vivo* studies and for what is possible, perform controlled trials for the most promising chemopreventive candidates, especially for defining the doses and recommendations of administration frequency. Although there are several epidemiological studies on the topic, most of them are not conclusive, mainly for being retrospective making it difficult to recall specific diet details. This is opposite to what is observed in *in vitro* studies, which is what makes controlled trials so necessary. That would be the ideal way of filling the gaps in this field when it comes to *in vivo* effects.

## Author Contributions

FM and WM designed the review. FM, JO, VS and WM selected and discussed articles. JO and VS prepared tables and figures. JO and VS wrote specific sessions. FM and WM wrote most of the article.

### Conflict of Interest Statement

The authors declare that the research was conducted in the absence of any commercial or financial relationships that could be construed as a potential conflict of interest.
